# Gene expression profiling suggests a pathological role of human bone marrow-derived mesenchymal stem cells in aging-related skeletal diseases

**DOI:** 10.18632/aging.100355

**Published:** 2011-07-28

**Authors:** Shih Sheng Jiang, Chung-Hsing Chen, Kuo-Yun Tseng, Fang-Yu Tsai, Ming Jen Wang, I-Shou Chang, Jiunn-Liang Lin, Shankung Lin

**Affiliations:** ^1^ National Institute of Cancer Research, National Health Research Institutes, Zhunan, Miaoli, Taiwan; ^2^ Division of Biostatistics and Bioinformatics, Institute of Population Health Sciences, National Health Research Institutes, Zhunan, Miaoli, Taiwan; ^3^ Institute of Cellular and System Medicine, National Health Research Institutes, Zhunan, Miaoli, Taiwan; ^4^ Orthopaedics Medicine, Miaoli General Hospital, Miaoli city, Taiwan; ^5^ Graduate Institute of Basic Medical Science, China Medical University, Taichung, Taiwan

**Keywords:** human bone marrow mesenchymal stem cell, aging, osteoporosis, osteoarthritis

## Abstract

Aging is associated with bone loss and degenerative joint diseases, in which the aging of bone marrow-derived mesenchymal stem cell (bmMSC) may play an important role. In this study, we analyzed the gene expression profiles of bmMSC from 14 donors between 36 and 74 years old, and obtained age-associated genes (in the background of osteoarthritis) and osteoarthritis-associated genes (in the background of old age). Pathway analysis of these genes suggests that alterations in glycobiology might play an important role in the aging of human bmMSC. On the other hand, antigen presentation and signaling of immune cells were the top pathways enriched by osteoarthritis-associated genes, suggesting that alteration in immunology of bmMSC might be involved in the pathogenesis of osteoarthritis. Most intriguingly, we found significant age-associated differential expression of *HEXA*, *HEXB*, *CTSK*, *SULF1*, *ADAMTS5*, *SPP1*, *COL8A2*, *GPNMB*, *TNFAIP6*, and *RPL29*; those genes have been implicated in the bone loss and the pathology of osteoporosis and osteoarthritis in aging. Collectively, our results suggest a pathological role of bmMSC in aging-related skeletal diseases, and suggest the possibility that alteration in the immunology of bmMSC might also play an important role in the etiology of adult-onset osteoarthritis.

## INTRODUCTION

Adult skeleton constantly undergoes bone remodeling to replace old/damaged bones by new bones, which is required for the maintenance of bone shape and strength of adult skeleton. However, bone mass decreases and bone fragility increases with age in both men and women. Although an increase in bone resorption rate associated with menopause is the primary cause of low bone mass in postmenopausal women, a decline in bone formation rate may also contribute to the loss of bone mass in both postmenopausal and age-related osteoporosis.

In bone marrow, mesenchymal stem cell (bmMSC) is a small population of multipotent cells that are capable of self-renewal proliferation and differentiating into several cell lineages such as osteoblast, chondrocyte, and adipocyte [1]. Thus, bmMSC play a critical role in bone formation that occurs in the skeletal development and growth, in the maintenance of fully grown skeleton, and in fracture repair [2]. It is reasonable to assume that the osteogenic potential of bmMSC may decrease with age. Indeed, animal studies have shown that bmMSCs from aged rats are less responsive to growth factors than cells from adult rats, and that bmMSCs from old bone are defective in bone induction potential [3-6]. However, the effect of aging on human bmMSC is not clear, and the pathological role of bmMSC in the aging-related bone defects is still under debate. An understanding of the changes in gene expression of bmMSC with age may provide clues and give insights into the basic cause of bone defects during aging.

In this study, using Illumina bead chip expression microarray, we analyzed the gene expression profiles of bmMSC derived from 14 donors between 36 and 74 years old, including many patients with osteoarthritis (OA). We identified genes whose change in expression was highly associated with age or OA. Putative biological functions and molecular pathways corresponding to those identified genes were retrieved by bioinformatics analysis, through which a possible pathological role of bmMSC in the development of skeletal diseases in aging was proposed.

### Patients and Methods

#### Isolation of bmMSC from bone marrow

Human bone marrows were either collected from osteoarthritis patients undergoing total knee replacement, or from the femurs of healthy donors receiving bone surgery because of trauma. Informed consent was obtained from each donor. The use of human bmMSCs in this study was approved by both the Institutional Review Board at National Health Research Institutes and Miaoli General Hospital. Bone marrows were subjected to ficoll density fractionation to collect the plastic-adherent mononuclear cells as described previously [7]. These cells were maintained in low glucose Dulbecco's modified Eagle's medium (GIBCO-BRL, California, USA) containing 20% fetal bovine serum (FBS) (Hyclone, Utah, USA), glutamine (GIBCO-BRL, California, USA), penicillin and streptomycin (GIBCO-BRL, California, USA), and maintained in a humidified atmosphere containing 5% CO_2_ at 37°C. After 10-14 days, colonies were dispersed and seeded at a density of 1.3 x 10^3^ cells/cm^2^ (passage 1), and were passaged at 70%-80% confluence afterward. Cells of the fourth passage were maintained in medium containing 10% FBS for 4-7 days and subjected to flow cytometric analysis. Cultures that were CD31- and CD45-negative, but CD90- and CD105-positive were defined as bmMSCs and used for RNA extraction [7].

#### RNA extraction, microarray experiments, and RT-qPCR validation

Total RNA was isolated from cells using Trizol (Invitrogen, Califonia, USA) according to the protocol provided by the manufacturer. Methods for sample labeling, array experiments, and TaqMan probe-based RT-qPCR experiments, were as described elsewhere [8]. The probes and primers used in RT-qPCR are as listed in Supplemental Table SIV.

#### Statistical analysis

(1) Data normalization. To avoid possible unwanted technical variation between samples or batches of array, raw data from microarray experiments were subject to either quantile normalization [9] using preprocessCore package in the Bioconductor, or global normalization that linearly adjust signal intensity according to signal of those spike-in control probes in the Illumina arrays (see supplemental methods). Normalized data were subjected to the following statistical analysis.

(2) Neighborhood analysis. This analysis, developed by Golub et al. [10], was used to detect if there were genes, in our array data, showing strong correlation in their expression with age or OA. This analysis was performed as described in supplemental methods.

(3) Multiple regression/ criteria of probes selection. Multivariate linear regression was used to model the expression level of a gene and its relation with age, gender, or the presence of OA of each subject. This model is described as follows:

For probe, *j* = 1,…,*J*, let *Y_ij_* be expression data of *i*th individual. For *i* = 1,…,*N*, let *A_i_*, *G_i_* and *D_i_* be the age, gender and OA respectively. In particular, *G_i_* = 1 if and only if the *i*th individual is male; *D_i_* if and only if the *i*th individual has OA. Assume the gene expression

$$Y_{ij}=\mu_{j}+\alpha_{A}A_{i}+\alpha_{D}D_{i}+\alpha_{G}G_{i}+\varepsilon_{ij}$$

where *α_A_*, *α_D_*, *α_G_* and *μ_j_* are the model parameters and *ε_ij_* is the error. Finally, a filter was applied to remove probes/genes having (i) average expression level < 300 arbitrary units or (ii) *p*>0.05 in multiple regression analysis, which then generated age-or OA-associated gene list. Since two normalization methods were applied, qualified probes/genes from either method that meet the above criteria were included in the candidate gene lists.

#### Measurement of DNA synthesis

DNA synthesis was assessed by measuring the incorporation of 5-bromo-2-deoxyuridine (BrdU) into DNA using BrdU Cell Proliferation Assay kit based on the protocol provided by manufacturer (Millipore, MA, USA). Briefly, cells were treated with BrdU or phosphate buffer saline (PBS, as background control) for 24 h. Cells were then fixed and DNAs were denatured using Fixing Solution. The BrdU label was detected by an anti-BrdU monoclonal antibody, and the signals were quantitated with a spectrophotometer microplate reader set at wavelength of 450/550 nm.

#### Bioinformatic analysis

We perform gene ontology and pathway analysis using Ingenuity Pathway Analysis 9.0 for enrichment of biological functions and pathways in which our selected genes of interest were involved.

## RESULTS

### 

#### Age-dependent differences in the growth rate of primary cultures of bone marrow-derived plastic-adherent cells

We analyzed the gene expression profiles of bmMSC derived from 14 human donors. Donors 1, 5, and 7 showed no signs of bone diseases and were designated as healthy donors, whereas the other 11 donors were osteoarthritis (OA) patients (Table I).

**Table I:** Demography of bone marrow donors.

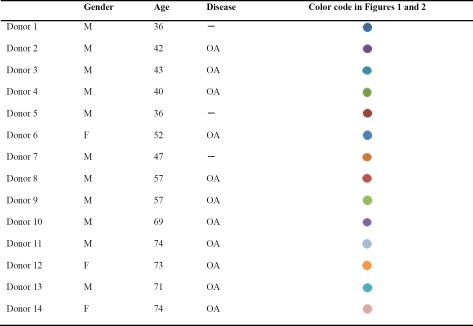

— means there is no sign of skeletal diseases

**Figure 1:**
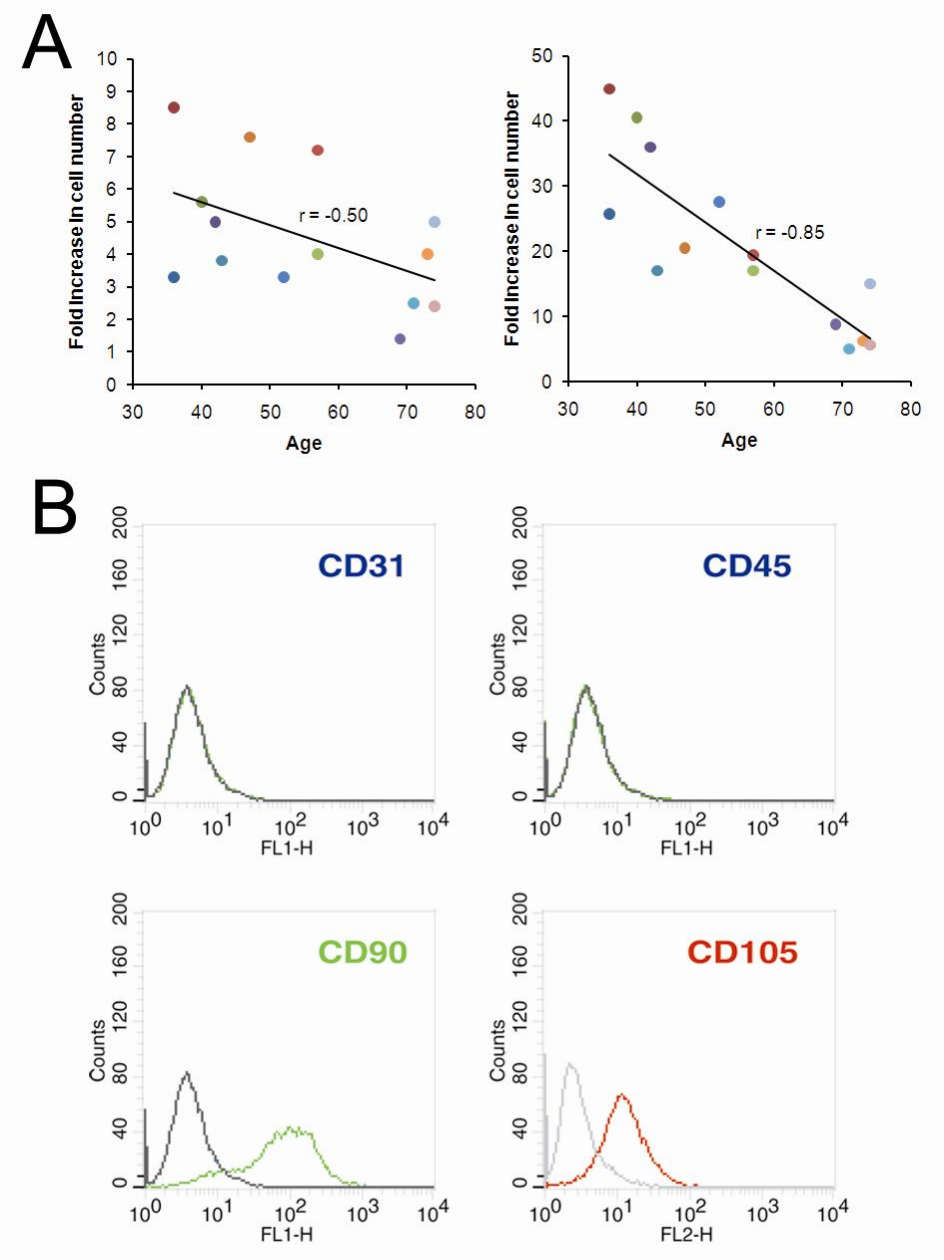
Growth rate of human bone marrow-derived plastic-adherent cells. (**A**) Plastic-adherent cells harvested separately from bone marrows of 14 donors were seeded at a density of 1.3 × 10^3^ cells/cm^2^ (passage 1). Cells were counted after 3 days (left panel) and 6 days (right panel) post-seeding using a hemocytometer, and the fold increase in cell number was calculated. Donors are color-coded as shown in Table I. (**B**) Cells harvested 6 days post-seeding were subjected to flow cytometric analyses. A representative result is shown.

While expanding stem cell populations from each bone marrow sample, we counted the plastic-adherent cells after three and six days post-seeding at passage one, calculated the fold increase in cell number for each line of bone marrow-derived cells, and correlated the data with the age of their donors. As shown in the left panel of Figure 1A, after three days post-seeding, the fold increase in cell numbers was negatively correlated with the age of donors, although this was not significant (*r*=−0.50, *p*=0.069). Nevertheless, a rather significant correlation (*r*=−0.85, *p*=0.001) was found after six days post-seeding (right panel, Figure 1A). Subsequently, these cells were characterized as bmMSC by flow cytometric analyses (Figure 1B).

#### Quality assessment of genome-wide microarray data and generation of age- and OA-associated genes

We noted that under *in vitro* culture condition, the gene expression profile of bmMSC might change with serial passaging. Indeed, it has been shown that *in vitro* aging has a similar effect as *in vivo* aging on human stem cells in terms of gene expression profiling [11]. Therefore, in this study, all the bmMSC cultures used for the genome-wide cDNA microarray analysis were harvested at the same passage (passage 4). It should be noted that clonogenic bmMSC is a heterogeneous mix of cells containing the multipotent stem cells and their progenitors. For convenience, these stem and progenitor cells were both named as bmMSC in this study.

**Figure 2:**
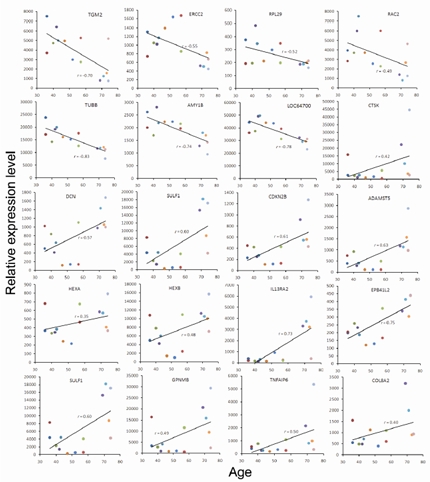
Representative plots of donor age *versus* normalized mRNA expression level for selective age-associated genes. Each solid dot represented a bone marrow donor. The regression lines (solid line) and correlation coefficients (*r*) showed trend of change in gene expression with increasing donor age. Donors are color-coded as shown in Table I.

The microarray analysis was performed using Illumina gene expression chips. Considering that the numbers of female donor as well as OA-free donor were low in this study, we thought that it would be more proper for us to focus on the age-related genes. However, because OA is an aging-associated disease, we also examined the gene expression profile associated with OA. First, we performed neighborhood analysis applying *t*-statistics (see Methods) to know if there exist genes that are associated with age or the presence of OA. The results showed that the expression level of many genes did have strong correlation with either age or the presence of OA (supplemental information, Figure S1-A and -B). Subsequently, we analyzed the microarray data with a multivariate linear regression model considering age, gender, and disease status (with or without OA) as covariates. Through such analysis, out of 48804 probes, the expression of 574 probes that contain 497 genes was found significantly correlated with age (*p*<0.05), as represented by 20 selected genes including *HEXA*, *HEXB*, *CTSK*, *SULF1*, *ADAMTS5*, *SPP1*, *COL8A2*, *GPNMB*, *RPL29*, *TNFAIP6*, *CDKN2B*, etc. (Figure 2, see Discussion). These genes were named age-associated genes (supplemental Table SI). On the other hand, there were 112 probes containing 92 genes correlated with OA, which were named OA-associated genes (supplemental Table SII). There was a moderate overlap of only 38 probes (29 genes) between age- and OA-associated genes (supplemental Table SIII).

We performed real-time quantitative PCR (RT-qPCR) analyses on 18 genes of interest selected from the abovementioned age- or OA-associated genes using cDNA prepared from selected adult and aged donors, and compared the results with array data. We calculated the correlation coefficients (*r*) between array and RT-qPCR data, and found that 11 of them had *r* >0.9 and 16 of them had *r* >0.8 (Table II). These results indicated that, in general, the results of RT-qPCR measurements were highly comparable with the array data.

**Table II:** Analysis of correlation between microarray data and RT-qPCR measurements.

Gene	*r*	*p*	Gene	*r*	*p*
*S100A4*	0.98	0.0006	*TGM2*	0.954	0.003094
*GPNMB*	0.998	4.78E-06	*STC1*	0.951	0.003594
*CDH6*	0.948	0.004	*NEFM*	0.371	0.469
*RRAGD*	0.888	0.017977	*TPI1*	0.869	0.024519
*CD55*	0.904	0.013265	*ADAM19*	0.935	0.006163
*CDKN2B*	0.971	0.00126	*RAC2*	0.859	0.028484
*NBL1*	0.928	0.0008	*AVPI1*	0.528	0.282
*SULF1*	0.859	0.028523	*KRT19*	0.948	0.004054
*PPFIBP2*	0.888	0.018161	*PDE1A*	0.966	0.001742

#### Gene Ontology/pathways analysis of age- or OA associated gene expression changes

**Figure 3:**
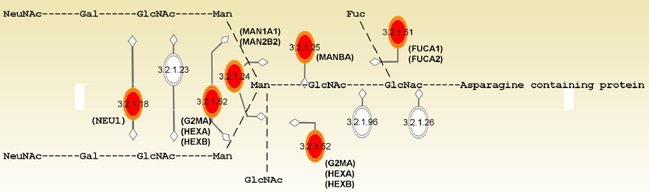
N-glycan degradation pathway enriched by pathway analysis. Enzymes (in EC number) with their names of coding genes and the corresponding sites of actions in the modification of N-glycan are indicated. Genes (n=9) that are differentially expressed with age and enriched by IPA analysis are marked in red.

To know what cellular functions or molecular pathways in which the age- and OA-associated genes were involved, we categorized these genes using the Ingenuity Pathway Analysis (IPA). Table III summarized the enriched top functions and canonical pathways. As shown, among the top ranked Gene Ontology function terms enriched by age-associated genes were cell growth and proliferation (*p* = 2.7×10^−5^~1.9×10^−2^), cellular movement (*p* = 2.94×10^−5^~2.07×10^−2^), cell cycle (*p* = 6.2×10^−5^~2.2×10^−2^), and cell morphology (*p* = 8.2×10^−5^~1.7×10^−2^). These results suggested that these cellular functions are most likely to change with age in human bmMSC. In spite of these results, there was no significant enrichment of specific cellular location (data not shown), indicating that the aging-related cellular activities might take place in various cellular compartments, rather than specific locations. Most intriguingly, our results showed that pathways for degradation of N-glycans (*p* = 8.9×10^−8^, Figure 3), degradation of glycosaminoglycans (GAGs, *p* = 5.7×10^−4^), and biosynthesis of glycosphingolipids (*p* = 5.7×10^−3^) were the top canonical pathways enriched by age-associated genes. The ratio of enrichment of genes in a specific pathway was 9/31, 6/71, and 4/45, respectively.

**Table III:** Top GO function terms and canonical pathways enriched by IPA.

	Top functions	p value	Top pathways	p value
Age-associated	Genetic Disorder	2.91E-06-2.01E-02	N-Glycan Degradation	8.91251E-08
	Metabolic Disease	1.1E-05-1.64E-02	Glycosaminoglycan Degradation	0.00057544
	Cellular Growth and Proliferation	2.66E-05-1.9E-02	Glycosphingolipid Biosynthesis - Globoseries	0.005754399
	Cellular Movement	2.94E-05-2.07E-02	Hepatic Fibrosis / Hepatic Stellate Cell Activation	0.00724436
	Cell Cycle	6.62E-05-2.15E-02	PTEN Signaling	0.011481536
	Cell Morphology	8.24E-05-1.68E-02	Glycosphingolipid Biosynthesis - Ganglioseries	0.012302688
	Cellular Development	8.24E-05-1.96E-02	Agrin Interactions at Neuromuscular Junction	0.013803843
	Skeletal and Muscular System Development and Function	1.18E-04-1.96E-02	Aminophosphonate Metabolism	0.013803843
OA-associated	Nucleic Acid Metabolism	5.26E-04-4E-02	Antigen Presentation Pathway	1.28825E-06
	Small Molecule Biochemistry	5.26E-04-4E-02	Crosstalk between Dendritic Cells and Natural Killer Cells	8.70964E-05
	Cardiovascular System Development and Function	8.96E-04-3.51E-02	Allograft Rejection Signaling	0.000933254
	Cell Morphology	8.96E-04-4.48E-02	Cytotoxic T Lymphocyte-mediated Apoptosis of Target Cells	0.001348963
	Cell-To-Cell Signaling and Interaction	8.96E-04-4.97E-02	OX40 Signaling Pathway	0.001862087
	Cellular Development	8.96E-04-4.97E-02	Cdc42 Signaling	0.003388442
	Nervous System Development and Function	8.96E-04-4.97E-02	Communication between Innate and Adaptive Immune Cells	0.006025596
	Cellular Assembly and Organization	2.23E-03-4.97E-02	Dendritic Cell Maturation	0.006309573

In addition, we found that nucleic acid metabolism (*p* = 5.3×10^−4^~4.0×10^−2^), small molecule biochemistry (*p* = 5.3×10^−4^~4.0×10^−2^), cell morphology (*p* = 9.0×10^−4^~4.5×10^−2^), and cell-to-cell signaling and interaction (*p* = 9.0×10^−4^~5.0×10^−2^) were the top ranked Gene Ontology function terms, while antigen presentation pathway (*p* = 1.3×10^−6^), cross talk between dendritic cells and natural killer cells (*p* = 8.7×10^−5^), and allograft rejection signaling (*p* = 9.3×10^−4^) were the top canonical pathways enriched by the OA-associated genes (Table III). The ratio of enrichment for each corresponding pathway was 5/43, 5/98, and 3/97, respectively. These results indicated a significant association between OA and bmMSC with altered immunological functions. Moreover, analyses showed that cell-mediated immune response, immunological disease, cell growth and proliferation, and cell cycle were among the top networks enriched by OA-associated genes (data not shown). Therefore, like increasing age, OA might also associate with alteration in the proliferative capacity of bmMSC. On the other hand, we found none of Gene Ontology function terms and pathways was enriched significantly by those 29 overlapping genes.

#### Analysis of the effect of sodium sulfate on the DNA synthesis in bmMSC from adult and aged donors

**Figure 4:**
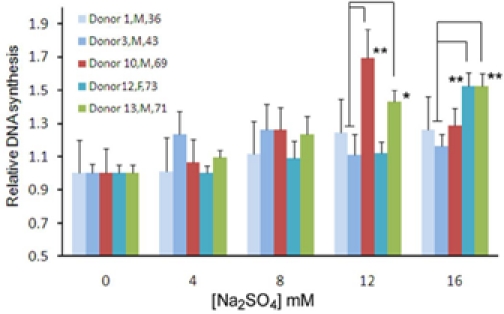
DNA synthesis measured by BrdU incorporation assays. BmMSC from donors 1, 3, 10, 12, and 13 were seeded into 96-well culture plate (1.2 × 10^2^ cells/well). Cells were either left untreated or treated with 4, 8, 12, and 16 mM Na_2_SO_4_ (Merck, Darmstadt, Germany) for 48 h. Then, either BrdU or PBS was added in medium, and cells were incubated for 24 h. Subsequently, cells were subjected to detection of the BrdU signals. The fold increase in DNA synthesis was calculated by comparing the BrdU signals of Na_2_SO_4_ treated cells to that of untreated cells (to which a value of 1 was assigned). The gender and age of donors are as indicated in the figure. Data represent the mean ± S.D. from three duplicate analyses. **, *p*<0.05; *, *p=*0.051 (comparison of individual aged sample to the average of adult samples, *t*-test).

As shown in supplemental Table SI, the expression of several sulfatase-encoding genes such as *SULF1*, *ARSB*, *IDS* in bmMSC were up-regulated with donor age. Sulfatases can desulfate the sulfated proteoglycans at the cell surface, affecting membrane metabolism, signal transduction, proliferation, etc, whereas, sodium sulfate (Na_2_SO_4_) increases sulfation of proteoglycans [12]. Since elevated levels of various types of sulfatases were found in aged donors and aged bmMSCs grew slower in culture, we tested whether attenuation in desulfation would be able to increase DNA synthesis differentially in bmMSCs from aged versus adult donors. Therefore, we treated adult (donors 1, 3) and aged (donors 10, 12, 13) bmMSCs with Na_2_SO_4_ of various doses and measured DNA synthetic activity by BrdU incorporation assays (Figure 4). For bmMSCs from donor 1, 12 mM Na_2_SO_4_ induced approximately 20% (*p*<0.01) increase in DNA synthesis, whereas 16 mM Na_2_SO_4_ did not further increase DNA synthesis. For bmMSCs from donor 3, Na_2_SO_4_ seemed not to induce DNA synthesis. In the aged group, for bmMSCs from donor 10, 12 mM Na_2_SO_4_ induced approximately 70% (*p*=0.013) increase in DNA synthesis. For bmMSCs from donor 12, 16 mM Na_2_SO_4_ induced approximately 60% (*p*=0.01) increase in DNA synthesis, whereas the increase induced by lower Na_2_SO_4_ concentration did not reach to statistical significance. For bmMSCs from donor 13, 8, 12, and 16 mM Na_2_SO_4_ induced approximately 30% (*p*=0.041), 40% (*p*=0.0014), and 50% (*p*=0.0013) increase in DNA synthesis, respectively. Moreover, the induction in DNA synthesis in bmMSCs from 2 out of 3 aged donors was significantly stronger than that in cells from 2 adult donors at higher Na_2_SO_4_ concentration (12 and 16 mM), which coincided with our findings that several sulfatase-encoding genes in bmMSC were up-regulated with donor age. Taken together, these results indicated that aged bmMSCs were more responsive to Na_2_SO_4_ treatment than adult bmMSCs in terms of induction in DNA synthesis, which supported the notion that elevated expression of sulfatases might cause a deleterious effect to the proliferative activity of bmMSCs from aged donors.

## DISCUSSION

Aged bones are featured by decreased bone mass and increased fragility compared with young bones. Inadequate bone formation following excessive bone resorption is a major cause of age-related bone loss. Besides, aged skeleton is also commonly accompanied by inflammatory disease such as OA, as represented by the bone marrow donors in this study. Given that bmMSCs give rise to bone-forming cells, it is likely that the aging of bmMSC play an important role in the aging of skeleton, and may be even involved in the development of aging-associated skeletal diseases such as osteoporosis (OP) and OA. Unfortunately, molecular evidence that can support the above conjecture and link aging of bmMSC to OP/OA is still lacking. It is thus of great interest to understand the age-associated gene expression change to know the role of bmMSC in the pathogenesis of aging-related skeletal diseases. In this regard, we examined the transcriptome-wide changes of genes of bmMSCs derived from 14 donors of various age, and analyzed the array data by exploiting a multivariate linear regression model considering all known variables, i.e., age, presence of OA, and gender. This analytic platform allowed us to correct effects of the OA- and/or gender-related change of gene expression, to obtain a list of age-dependent genes (in the background of OA), and also a list of OA-associated genes (in the background of old age). As far as the age-associated genes are concerned, our data are in agreement with the results reported by Wagner et al. who demonstrated related effects of aging and replicative senescence on the gene expression profiles of human bmMSC/progenitors [11]. However, there is an overlap of only 8 genes between our and their data. These include the age-associated up-regulation of *EPB41L3 and TCEAL7* involved in regulating cell proliferation; *IL13RA2* involved in the signaling of transforming growth factor β1-mediated fibrosis; *MFAP5* encoding a microfibrillar associated protein; *ROBO1* involved in axon guidance; *S100A4* encoding a calcium binding protein; *STEAP3* involved in iron metabolism; and *UBE2E2* involved in protein degradation. The little overlap might be due in part to the differences in the populations studied, cell culture conditions, array data processing, or analytical platform used. In addition to the above mentioned genes, we report novel findings about the age- and OA-associated changes in the gene expression profiles of bmMSC.

Our data point out that cell growth, proliferation, and migration are the cellular functions that most possibly change with age in human bmMSC. Among the genes involved in these cellular functions are the cell cycle regulators-encoding *CCND2*, *CCNE1*, and *CDKN2B*. The former two genes encode D- and E-type cyclins, whereas *CDKN2B* encodes a cyclin-dependent kinase (CDK) inhibitor p15^INK4b^ which arrests cell cycle by inhibiting the D-type cyclin-dependent kinase CDK4 activity. We observed that *CCND2* and *CDKN2B* are up-regulated, but *CCNE1* is down-regulated with age. Given the findings that CDK inhibitors play an important role in regulating the renewal proliferation of mice hematopoietic stem cells [13-15], and that there is a strong link between p16^INK4a^ and cellular aging [13-16], our results suggest that regulation of *CDKN2B* expression may play an important role in the renewal proliferation as well as aging of human bmMSC. Besides the cell cycle regulators, we found the up-regulation of *DCN*, *PODN*, *TP53INP1*, and *DRAM1*, and down-regulation of *ERCC2* and *TGM2* with age. *DCN* encodes a proteoglycan which down-regulates the proliferation and migration of mammalian cells [17]. *PODN* encodes an extracellular matrix (ECM) component which inhibits cell growth and migration [18]. *ERCC2* encodes a nucleotide excision repair enzyme critical for removal of damaged DNA fragments, while *TP53INP1* and *DRAM1* participate in the DNA damage-triggered growth arrest and apoptosis [19, 20]. As for *TGM2*, it was found to enhance cell growth and survival through anti-apoptosis signaling [21]. Interestingly, *TGFBR3*, *RPS6KA2*, *PTGER4*, *FBXO32*, *SULF1*, *DBC1*, *TCEAL7*, and *EPB41L3* are up-regulated with age (supplemental Table SI). These genes have been reported to negatively control cancer cell proliferation [22-29]. Thus, up-regulation of these ‘tumor suppressor genes’ is likely to decrease the proliferation rate of human bmMSC. Data described above might underlie the aging-associated decrease in the proliferation rate of bmMSCs, an aging phenotype of mammalian bmMSCs [3, 30, 31]. Since our data are in agreement with the current findings regarding the deleterious effect of aging to the proliferation of stem cells, it is conceivable that our results can also reveal the other important age-associated functional changes in human bmMSC. Among them, as revealed by our analyses, are those involved in glycobiology.

Glycosylation is a cellular process that links glycans to macromolecules such as proteins and lipids by different types of glycosidic bonds. N-linked glycans (N-glycans), for example, are the polysaccharides that link to the peptides or proteins by N-glycosidic bond. Mature glycoproteins and glycolipids not only form the architecture of cell membrane but also participate in cellular signaling. Our results show that several genes involved in the modification of glycan are up-regulated with age (Table IV and Figure 3). Since modification of glycan is a pivotal process in the synthesis and catabolism of glycoproteins and glycolipids, up-regulation of these genes with age suggests that aging of human bmMSC may be accompanied by alterations in membrane homeostasis and in the glycosylation of membrane components, which may result in the alteration in cellular signaling. For example, hexosaminidase has been implicated in local hydrolysis of glycosphingolipids at cell membranes [32]. Given that glycosphingolipid is the major component of lipid rafts which play an important role in a variety of cellular processes including signal transduction and cell proliferation, elevated expression of *HEXA* and *HEXB* might enhance the degradation of glycosphingolipids at aged bmMSC surface, impact the formation of lipid rafts, and affect signaling. For another example, sulfatase 1 is able to desulfate the sulfated proteoglycans at the cell membrane, inhibits their co-receptor functions in the signaling of several growth factors. Accordingly, elevated expression of *SULF1* in aged bmMSC is likely to impair cellular response to growth factors. In fact, we show that Na_2_SO_4_ is able to induce DNA synthesis in bmMSCs from adult and aged donors, and the induction is stronger in bmMSCs from aged donors than in bmMSCs from adult donors (Figure 4). Based on these findings, we postulate that alterations in the cellular functions regulating membrane homeostasis and glycosylation of membrane components are very likely to alter the proliferative capacity of bmMSC, and play an important role in the aging of bmMSC.

**Table IV:** Genes involved in glycan modification

Gene symbol	Product	Function
*GLT8D2*	Glycosyltransferase 8 domain containing 2	glycosyltransferase
*FUCA1*	Tissue alpha-L-fucosidase 1	fucosidases
*FUCA2*	Tissue alpha-L-fucosidase 2	fucosidases
*MAN1A1*	Mannosidase, alpha, class 1A, member 1	mannosidases
*MAN2B2*	Mannosidase, alpha, class 2A, member 2	mannosidases
*MANBA*	Lysosomal mannosidase, beta A	mannosidases
*NEU1*	Lysosomal sialidase 1	Sialidase
*HEXA*	Hexosaminidase A (*α*-polypeptide)	hexosaminidase (glycosylhydrolase)
*HEXB*	Hexosaminidase B (β-polypeptide)	hexosaminidase (glycosylhydrolase)
*GM2A*	GM2 ganglioside activator	cofactor of hexosaminidase
*ARSB*	Arylsulfatase B	sulfatases
*IDS*	Iduronate 2-sulfatase	sulfatases
*SULF1*	Sulfatase 1	sulfatases

In addition, our results have provided clues to address the involvement of bmMSC in aging-associated skeletal diseases. OA is an inflammatory disease featured by the degeneration of cartilage matrix, which is due in part to excessive degradation of the matrix components aggrecan, collagen II and GAG [33-35]. It has been reported that hexosaminidase and sulfatase 1 which are involved in the degradation of GAG are the dominant enzymes in the synovial fluid and cartilage of OA patients [36, 37]. Inhibition of hexosaminidase activity has been proposed for preventing or even reversing cartilage degradation in OA patients [34]. ADAMTS5, an aggrecanase, was also found highly expressed in human OA cartilages [38]. Deletion of active *ADAMTS5* has been shown to prevent cartilage degradation in a murine OA model [39]. As to the degradation of collagen II, cathepsin K was found involved in the cleavage of collagen II in articular cartilages in certain OA patients, suggesting that it might play a role in OA pathology [33]. Our data show that genes encoding these enzymes in bmMSCs are all up-regulated with age. In addition, *COL8A2* and *GPNMB*, two OA candidate genes [40], are also up-regulated with age in bmMSCs. Given that bmMSCs are the primary source of cartilage chondrocytes, our data suggest a pathological role of aged bmMSC in aging-associated OA.

Moreover, the age-associated genes also cover genes participating in regulating bone resorption and formation. Data show that *RPL29* is down-regulated with age in human bmMSC. *RPL29* encodes a ribosomal protein. Mice lacking this gene display a short stature phenotype and exhibit increased bone fragility, which is due to delayed entry of osteoprogenitors into cell cycle and altered matrix protein synthesis rates [40]. In addition, we show that *TNFAIP6* is up-regulated with age. This gene has been found down-regulated during osteoblastic differentiation; overexpression of this gene inhibits osteoblastic differentiation of human MSCs [41]. Thus, down-regulation of *RPL29* and up-regulation of *TNFAIP6* with age may represent a mechanism underlying the aging-associated defects in bone formation and osteoblastic differentiation of human bmMSC. As mentioned above, cathepsin K may play a role in OA pathology, and is up-regulated with age. In fact, there are evidences showing that capthesin K is also implicated in the pathogenesis of OP: (i) cathepsin K has been considered as a target for the pharmacological treatment of OP, and (ii) overexpression of *CTSK* has been shown to cause spontaneous development of synovial hyperplasia and fibrosis, cartilage degeneration, and bone destruction in transgenic mice upon aging [42]. Therefore, it is conceivable that osteoprogenitors/osteoblasts with elevated expression of *CTSK* may jeopardize their osteogenic activity. With these in mind, it is not surprising to find that *SPP1*, an OP susceptibility gene [43], is up-regulated with age in bmMSC (supplemental Table SI). Thus, our findings provide compelling molecular evidences to suggest a role of aged bmMSC in the pathogenesis of OP. Meanwhile, it has to be mentioned that there is a moderate overlap between age- and OA-associated gene lists though, the age-associated genes discussed above are only present in the former gene list. Taken together, it is tempting to postulate that by associating with the forming of pathological gene expression profile described above, increase of age may act as an intrinsic promoting factor to the development of aging-associated skeletal diseases.

Our analyses of the OA-associated genes have shown interesting findings regarding the etiology of OA. Based on current theory, OA is the consequence of long term mechanical stress on the articular cartilage. In response, the cartilage chondrocytes produce inflammatory cytokines and matrix metalloproteinases, which eventually causes destruction of articular cartilage. But recently, a genome-wide association study (GWAS) identified two single nucleotide polymophisms (SNPs) which are in a region containing HLA class genes including *HLA-DRB4*, associated with susceptibility to knee OA [44]. This finding suggests that immunological mechanism may be implicated in the etiology of OA. Here, we show that antigen presentation and signaling of immune cells are the top pathways enriched by OA-associated genes, and that *CD74* and a list of *HLA* class genes including *HLA-DRB4* are down-regulated with OA (Table III). Thus, coinciding with that GWAS result, our results also suggest an immunological issue associated with OA. Accordingly, we propose that bmMSC with altered immunological property might play an important role in the etiology of OA. On the other hand, we found that *DAXX* which encodes a pro-apoptotic factor in primary cells [45] is up-regulated with OA. Oppositely, *GAS6*, *SKI*, and *RAD51* are down-regulated with OA (supplemental Table SII). Gas6 can promote cell proliferation, survival, and migration [46]. Ski can bind to the histone deacetylase SIRT1 and inactivate p53 [47]. Rad51 is the major recombinase involved in the repair of DNA double strand breaks. So, our results suggest that the presence of OA might associate with deficient DNA repair, and decreased proliferation and survival of bmMSC.

In summary, we have reported novel findings regarding to the age- and OA-associated changes in the gene expression profiles of human bmMSC. We show that increase of age and the presence of OA may independently associate with changes in gene expression profile that may hinder the proliferation and survival of bmMSC. In particular, our results suggest a pathological role of aged bmMSC in the development of OP and/or aging-associated OA, and also suggest a role of bmMSC with altered immunological property in the etiology of ‘adult-onset’ OA.

## SUPPLEMENTAL MATERIAL

The Supplemental Information is found in Full Text version of this manuscript.

## Abbreviations

bmMSC: bone marrow-derived mesenchymal stem cell
OA: osteoarthritis
OP: osteoporosis
