# Supplementary material for: Gene expression profiling suggests a pathological role of human bone marrow-derived mesenchymal stem cells in aging-related skeletal diseases
Source: Aging (Albany NY). 2011 Jul 28;3(7):672–84. doi: 10.18632/aging.100355 (PMC3181167; doi:10.18632/aging.100355)

**Supplemental information**

**Note: full table of Table SI is available in MS Excel file format (TableSI.xlsx).**

**Note: full table of Table SII is available in MS Excel file format (TableSII.xlsx).**

**Note: full table of Table SIII is available in MS Excel file format (TableSIII.xlsx).**

| **Table SIV. Primers and probes used in RT-qPCR validation assay.** | | | | | |
| --- | --- | --- | --- | --- | --- |
| **Gene** | **Unigene ID** |  | **Primer-F** | **Primer-R** | **Universal Probe Library probe ID** |
| *S100A4* | NM_019554.2 |  | cttgcacacgctgttgctat | cgagtacttgtggaaggtgga | 81 |
| *GPNMB* | NM_002510.2 |  | acccaccccttctttaggac | tctggcagttttcatcagga | 1 |
| *PDE1A* | NM_001003683.1 |  | aaacacctttaagtaaaacctcgtg | aaaatctccaagtcttttggtca | 87 |
| *RRAGD* | NM_021244.4 |  | ggctagcggactacggaga | ggggtcactgaagtccagaac | 82 |
| *CD55* | NM_000574.3 |  | acccttggtgacgcagag | tggctccgcaataccagt | 26 |
| *CDKN2B* | NM_078487.2 |  | cagtcgatgcgttcactcc | ggcctaagttgtgggttcac | 73 |
| *SULF1* | NM_015170.2 |  | cagcctccaatccgtaagag | tcagcacagtggtgtgtcaa | 23 |
| *PPFIBP2* | NM_003621.2 |  | ctagtcatgcgctggaagc | ttttagtgcctgcaatgatcc | 1 |
| *TGM2* | NM_004613.2 |  | agacggtggagatcccaga | cagcaggtccattctcacct | 16 |
| *NEFM* | NM_005382.2 |  | tgccggctacatagagaagg | tctccgcctcaatctcctta | 1 |
| *STC1* | NM_003155.2 |  | tctgaaggaccccaggtaag | agagtagcctctgggttgagg | 14 |
| *TPI1* | NM_000365.5 |  | ctggcactaggtcttgtggtt | gctgcctcaaaaaggaagatt | 8 |
| *ADAM19* | NM_033274.2 |  | cctggatggacaagaggaag | tgaagtcttccactgaggtatgat | 8 |
| *RAC2* | NM_002872.3 |  | ctgcttgccagagagttcct | ttgttccaaaagaggagaactga | 82 |
| *CDH6* | NM_004932.2 |  | ttgctcaacatggatcgaga | atccttggcttgaatcacca | 32 |
| *GAPDH* | NM_002046.3 |  | ctctgctcctcctgttcgac | acgaccaaatccgttgactc | 60 |
| *KRT19* | NM_002276.4 |  | ataaaaggcgccaggtgag | agcaaccctggtctcagaag | 52 |
| *ACTIN* | NM_001101.3 |  | aagtcccttgccatcctaaaa | atgctatcacctcccctgtg | 55 |
| *AVPI1* | NM_021732.2 |  | ctggcccttgtaagcacct | cctcttccacagccttcaga | 4 |
| *NBL1* | NM_182744.2 |  | gtgtggagggtgcgagtag | catcctgggtggctccta | 40 |

**Supplemental methods**

*Microarray data normalization*

Two kinds of data normalization methods were applied in this study: (1) quantile normalization, using preprocessCore package (Bolstad *et al.* 2003) in the Bioconductor, and (2) spike-in controls-based global normalization. We now describe the latter normalization method as follows. In the Illumina Beadchips array, a series of spike-in controls that will show three- level (low, medium, and high) of hybridization signal intensities are included in the hybridization experiment of each sample. The experiment is considered a failure if the intensities of these controls are not in the expected pattern/order. We found that the intensities of the controls in each of the 14 samples revealed the expected pattern/order. The normalization makes use of these nicely behaved controls and performs an interpolation based on these controls. The procedure is detailed below. We denote the mean measurements of low controls, median controls and high controls in samples$h$by$a_{h}$,$b_{h}$and$c_{h}$respectively. Let$a$,$b$and$c$denote respectively the mean measurements of all the low controls, medium controls and high controls from all the samples in this study. The normalization procedure for genes of samples in sample$h$is accomplished by using the unique continuous piecewise linear function that maps$a_{h}$,$b_{h}$and$c_{h}$to$a$,$b$and$c$respectively. Namely, its value at$x$is

$$\left\{\begin{matrix} x-c_{h}+c \\ \left(a-c\right)\left(x-c_{h}\right)/\left(a_{h}-c_{h}\right)+c \\ \left(b-a\right)\left(x-a_{h}\right)/\left(b_{h}-a_{h}\right)+a \\ x-b_{h}+b \end{matrix}\right. \begin{matrix} \text{if }x\leq c_{h}; \\ \text{if }c_{h}<x\leq a_{h}; \\ \text{if }a_{h}<x\leq b_{h}; \\ \text{if }b_{h}<x. \end{matrix}$$

The expression level of genes of in sample$h$is to be replaced by the value of the piecewise linear function.

*Neighborhood analysis*

We showed that the observed correlations were stronger than would be expected by chance. This is established by neighborhood analysis developed by Golub *et al* (Golub *et al.* 1999). We ordered the bmMSC samples according to their donor age, with sample 1 and sample 14 being the youngest and oldest, respectively. Let group A be the samples numbered from 1 to 8 and group B those numbered from 9 to 14. We used *t*-statistics to compare the expression of a given gene g in samples between group A and group B. That is, each gene g defines a *t*-statistic, denoted by $t_{g}$, to compare the expression levels of gene g in group A and in group B. In addition, we performed 1000 random permutations among the sample order, so that after the permutation, samples in group A are not the younger ones anymore. Then, we repeated the t-statistics for each given gene g as described above. We calculated the $t_{g}^{i}$ which denotes the *t*-statistic for the comparison in the expression of a given gene g in group A and in group B after the $i^{th}$permutation. The number of genes with *t-*statistics greater than various threshold levels were calculated and plotted as shown in Figure S1A. There are three curves and each represents real condition (without permutation, black), 1^st^ percentile of permutated data (blue), and 5^th^ percentile of permutated data (red), respectively. Consider the intersections of these three curves with a given vertical line; for example, the vertical line passing through the point (3,0). The black dot has coordinate (3, 11530), which means that there are 11530 genes whose *t*-statistics $t_{g}$are greater than 3. The blue dot has coordinate (3, 1499), which means among the 1000 permutated datasets, one percent of them have more than 1499 genes having t-statistic greater than 3. The red dot gives the five percent point. Dots with negative first coordinate count the number of genes having t-statistics less than that negative number. Figure S1A strongly suggested that the number of genes that are strongly correlated with age is significantly larger than would be expected by chance. By similar analysis, we showed that the number of genes strongly correlated with presence of OA is also significantly larger than would be expected by chance (Figure S1B).

**Reference:**

T.R. Golub, D.K. Slonim, P. Tamayo, C. Huard, M. Gaasenbeek, J.P. Mesirov, H. Coller, M.L. Loh, J.R. Downing, M.A. Caligiuri, C.D. BloomfieldE.S. Lander, Molecular classification of cancer: class discovery and class prediction by gene expression monitoring, Science. 286 (1999) 531-537.

**Figure S1.**

A


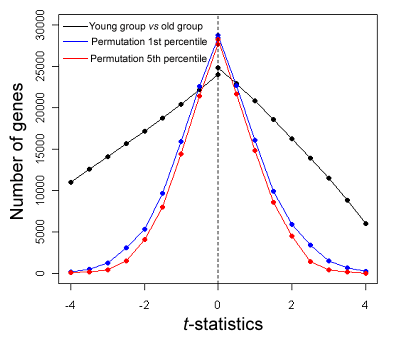


B


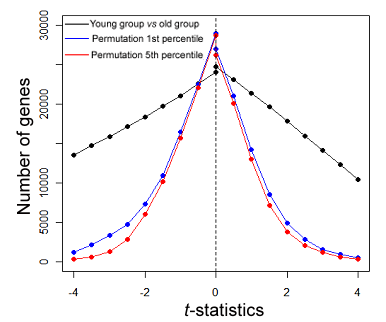

Supplement: Supplementary file 1 — The Supplemental Information is found in Full Text version of this manuscript. [file aging-03-672-s001.docx]
